# The Influence of Differently Shaped Gold Nanoparticles Functionalized with NIPAM-Based Hydrogels on the Release of Cytochrome *C*

**DOI:** 10.3390/gels3040042

**Published:** 2017-11-08

**Authors:** Sulalit Bandyopadhyay, Anuvansh Sharma, Wilhelm Robert Glomm

**Affiliations:** 1 Ugelstad Laboratory, Department of Chemical Engineering, Norwegian University of Science and technology (NTNU), N-7491 Trondheim, Norway; anuvansh.sharma@ntnu.no (A.S.); Wilhelm.glomm@sintef.no (W.R.G.); 2 Polymer Particle and Surface Chemistry Research Group, SINTEF Materials and Chemistry, N-7465 Trondheim, Norway

**Keywords:** anisotropy, gold, cytochrome *C*, NIPAm, nanostructures, PEG

## Abstract

Here, we report the synthesis and functionalization of five different shapes of Au nanoparticles (NPs), namely nanorods, tetrahexahedral, bipyramids, nanomakura, and spheres with PEG and poly (*N*-isopropylacrylamide)-acrylic acid (pNIPAm-AAc) hydrogels. The anisotropic NPs are synthesized using seed-mediated growth in the presence of silver. The NPs have been characterized using Dynamic Light Scattering (DLS), zeta potential measurements, UV-Visible spectrophotometry (UV-Vis), and Scanning Transmission Electron Microscopy (S(T)EM). Cyt *C* was loaded into the PEG-hydrogel-coated AuNPs using a modified breathing-in method. Loading efficiencies (up to 80%), dependent on particle geometry, concentration, and hydrogel content, were obtained. Release experiments conducted at high temperature (40 °C) and acidic pH (3) showed higher release for larger sizes of PEG-hydrogel-coated AuNPs, with temporal transition from spherical to thin film release geometry. AuNP shape, size, number density, and hydrogel content are found to influence the loading as well as release kinetics of Cyt *C* from these systems.

## 1. Introduction

Anisotropic gold nanoparticles (AuNPs) show remarkable optical properties such as localized surface plasmon resonance (LSPR) and fluorescence that have potential applications in bio-sensing [1] photothermal applications [2,3,4,5,6,7], targeted drug delivery [7,8,9,10], bioimaging [11,12,13], gene delivery [14,15,16], catalysis [17,18,19], and other biomedical applications. A large number of anisotropic AuNPs, namely nanorods, nanopyramids, nanotubes, nanocages, nanowires, and nanostars, have been reported to be used as biosensors and bioprobes [20,21,22]. AuNPs are highly promising for biomedical applications due to long body circulation times ranging from a few hours to days [23], and show selective accumulation at desired target sites resulting from the enhanced permeability and retention (EPR) effect or the surface functionalization with specific ligands [24,25,26], large absorption in the near-infrared (NIR) window for photothermal therapy, and facile surface functionalization. However, the prolonged blood and body half-life of NPs is primarily size-dependent. NPs with a hydrodynamic diameter (HD) of less than 5 nm are capable of clearance across the endothelium as well as glomerular filtration, whereas larger particles experience longer circulatory times [27]. Out of all the shapes, Au nanorods (AuNRs) have attracted the most interest. 

In addition to the shape, the surface coating determines potential applications of AuNPs. Although cationic functionalized AuNPs have been applied for drug delivery applications [14], positively charged NPs are considered to be particularly toxic as they can set off cascades that induce apoptosis and cause the production of reactive oxygen species [28,29]. Cetyltrimethylammonium bromide (CTAB)-coated anisotropic AuNPs have been synthesized previously by our group and others [30,31,32,33]. However, the presence of excess CTAB hinders serum proteins from adsorbing on the surface and alters the process of endocytosis [34]. Thus, removal of CTAB is essential in order to reduce the cytotoxicity of the NPs. In order to reduce the cytotoxicity from the cationic ligand, successful replacement of CTAB has been shown by using PEG [35] as well as successive PEG and mercaptoundecanoic acid (MUA) functionalization [36]. Researchers have also combined the optical properties of gold with stimuli-responsive properties of poly (*N*-isopropylacrylamide) (pNIPAm)-based hydrogels. Acrylic acid (AAc)-based pNIPAm hydrogels associated with AuNPs have been shown to provide pH response in addition to temperature sensitivity [37,38,39]. Research conducted by Lyon et al. has shown the potential of pNIPAm-based stimuli-responsive hydrogels as an emerging class of drug delivery vehicles [40,41]. 

The majority of functionalization studies include a comparison of the multifunctional properties of spherical AuNPs with Au nanorods or nanocages. To our knowledge, there have been no systematic studies that compare different anisotropic AuNPs synthesized using the same protocol in terms of their release properties of model protein drugs, after successful replacement of cationic surfactants. Although previous studies have shown successful functionalization of AuNPs with pNIPAm-AAc hydrogels, the effect of introducing anisotropy to such systems has not been investigated. The drug loading and release characteristics from functionalized AuNPs have mostly considered size as a parameter for comparison or spherical Au as a benchmark NP. However, shape anisotropy, coupled with coating thickness or available surface area for drug absorption within the AuNP systems, has not been studied.

Herein, we report a systematic functionalization study for five differently shaped AuNPs, namely spheres (AuNS), rods (AuNR), tetrahexahedral (AuHex), bipyramids (AuBP), and nanomakura (AuNM), with AuNM being reported previously by our group for the first time [42]. These NP have been coated with PEG followed by a pNIPAm-AAc-based hydrogel. Their size, charge, and optical properties have been mapped as a function of coating. The effect of size, shape, particle number, and hydrogel content has been studied on the loading and release kinetics of a model protein drug, Cytochrome *C* (Cyt *C*). 

## 2. Results and Discussion

**Figure 1:**
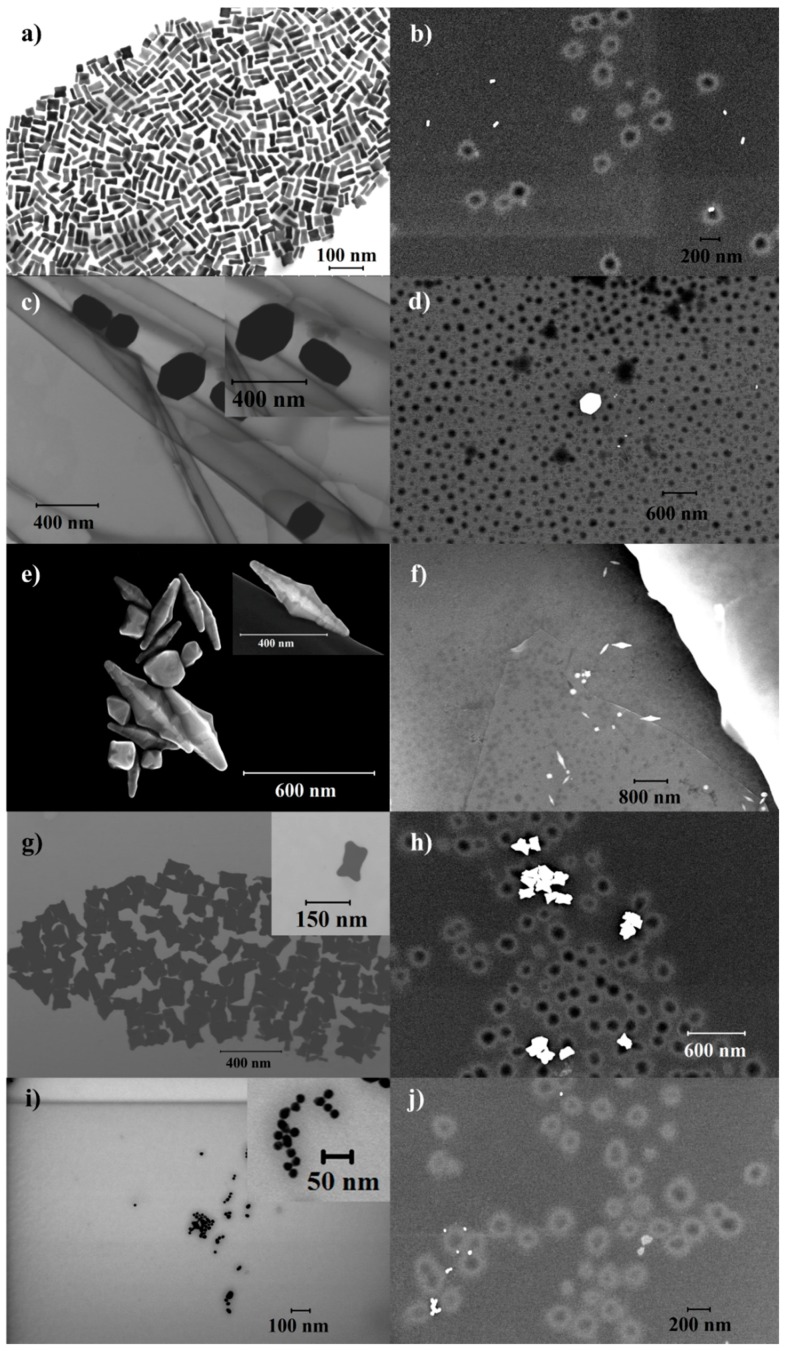
S(T)EM images of (**a**) AuNR; (**b**) PEG-hydrogel coated AuNR; (**c**) AuHex; (**d**) PEG-hydrogel coated AuHex; (**e**) AuBP; (**f**) PEG-hydrogel coated AuBP; (**g**) AuNM; (**h**) PEG-hydrogel coated AuNM; (**i**) AuNS; and (**j**) PEG-hydrogel coated AuNS.

The different anistropic shapes of the AuNPs used in the study were synthesized using an Ag-assisted, surfactant-mediated seeded growth method, while spherical NPs were synthesized using a modified Turkevich method. Figure 1a,c,e,g,i show the S(T)EM images of the various AuNPs, namely rods (AuNR), tetrahexahedral (AuHex), bipyramids (AuBP), nanomakura (AuNM) and spheres (AuNS). The same NPs, after successful two-step coating using PEG and hydrogel, were observed under S(T)EM and are shown in Figure 1b,d,f,h,j, respectively. It can be observed that the AuNPs are located between the hydrogel units for all the shapes. However, the S(T)EM images have been analyzed as complementary to DLS sizes to comment on the behaviors of the AuNPs and PEG-hydrogel-coated NPs in solution conditions.

**Figure 2:**
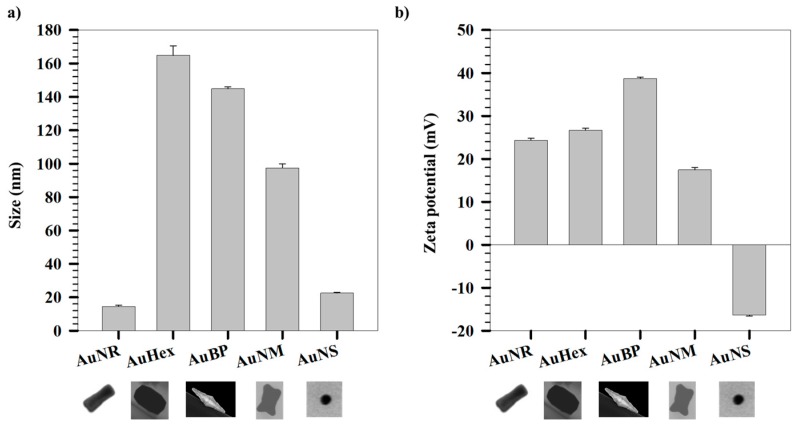
(**a**) Hydrodynamic sizes and (**b**) zeta potentials of different shapes of AuNPs.

Figure 2a,b show the sizes and zeta potentials of differently shaped AuNPs. DLS measures the hydrodynamic size based on the assumption that the equivalent spherical particle diffuses at the same rate as that of the parent particle whose size is being reported. The spherical NPs, namely AuNS, are found to have a hydrodynamic size of 22.5 ± 0.4 nm with a zeta potential of −16.4 ± 0.2 mV. The negative surface charge depicted by a negative zeta potential is indicative of citrate capping on these NPs. Among the NPs studied, the smallest NPs are nanorods, namely AuNR, having a hydrodynamic size of 14.4 ± 0.7 nm. The DLS results further show that the sizes of other anisotropic NPs decrease in the order hexagonal, bipyramids, and nanomakura (AuHex > AuBP > AuNM). However, the hydrodynamic sizes, when compared to the S(T)EM analysis, show that the largest NPs are the bipyramids (length: 382 ± 107, diameter: 107 ± 45), while the smallest are the spherical NPs (AuNS), having a size of 17 ± 2 nm (Figure 1). 

The differences can be attributed to measurement principles and the diameters being reported. While DLS measures hydrodynamic radius based on scattering due to the Brownian motion of suspended particles in solution, S(T)EM measures the dry radius based on the electrons transmitted through a thin slice of the sample. The hydrodynamic radius is based on the assumption that the particles are spherical in shape and also accounts for ligands bound to the surface of the particle. In the case of the differently shaped NPs studied here, the particles have multiple axes of rotation owing to shape anisotropy. This means that the DLS data may not be interpreted singly to understand the geometries of the particles and their size-based properties. Table 1 shows the sizes of the different AuNPs measured using S(T)EM. It can be observed that the anisotropic NPs show larger sizes compared to the DLS hydrodynamic sizes, while the sizes of the spherical NPs show close agreement with their hydrodynamic radius. A slight increase in the DLS size is observed due to measurements of different diameters; DLS measures the hydrodynamic radius rather than the core radius of the NPs as measured by S(T)EM, with a weak interparticle dipolar interaction among NPs causing weak interparticle coupling, or a combination, as reported previously [43].

The aspect ratios (ratio of length to the particle diameter) of AuNRs (3.4 ± 0.5) is comparable to that of AuBPs (3.8 ± 0.5), although their sizes differ considerably. A similar trend is noted for AuHex and AuNM, having ARs of 1.4 ± 0.2 and 1.5 ± 0.4, respectively. This variation in sizes has also been observed previously by our group [42]. The anisotropic AuNPs show positive zeta potentials owing to the cationic CTAB coating on the NPs. Zeta potential, which is a measure of colloidal stability, is measured based on the electrophoretic mobility of charged particles in solution subjected to an electric field. The high values of zeta potentials for all the systems indicate colloidal stability under the measurement conditions. However, cationic NPs absorb or bind strongly to lipid bilayers constituting the cell membrane, resulting in deformation of the membrane and subsequent cytotoxic effect on the cell [44,45,46]. The effect of cytotoxicity due to cationic charge from the CTAB layer has been shown to be reduced by replacement of CTAB with other ligands that have stronger adsorption preference for gold, which also reduces the cationic charge on the NPs [47]. Herein, the CTAB layer has been subsequently reduced by sequential coating with PEG followed by hydrogel. The PEG layer provides stealth behavior to the NPs owing to the hydrophilic nature of PEG, which prevents opsonization of plasma proteins on the NP surface [48]. In addition to behaving as a cloaking polymeric shell, PEG increases the circulation half-life of NPs delivered systemically in the blood [49]. On the other hand, the pNIPAm-AAc-based hydrogel layer imparts stimuli-responsive properties as a function of temperature and/or pH, as has been shown in our previous work [50].

**Figure 3:**
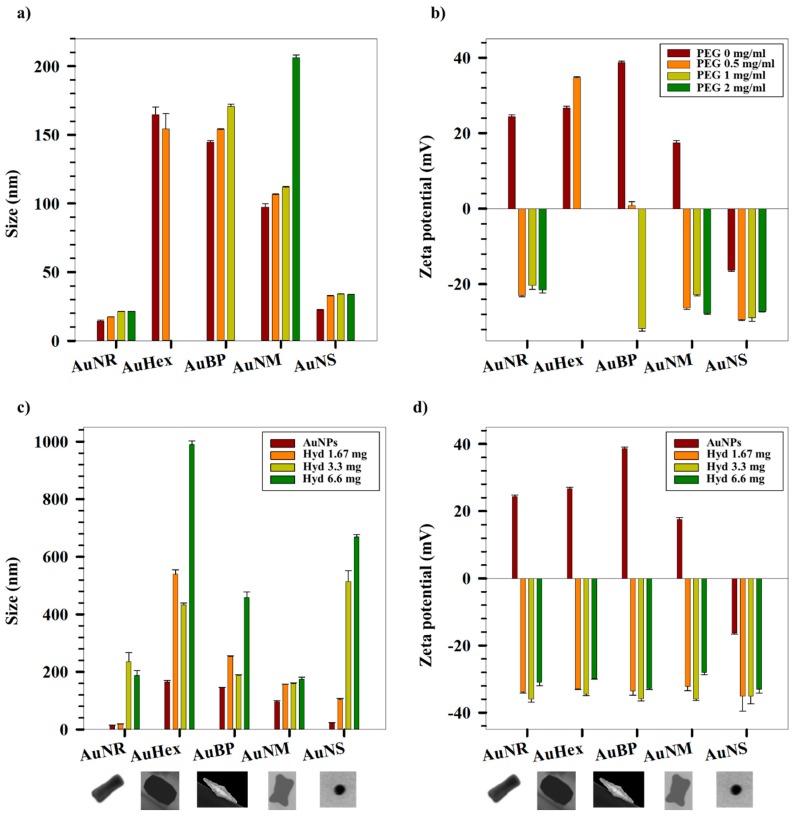
Variation of (**a**) hydrodynamic sizes and (**b**) zeta potentials of AuNPs with different concentrations of PEG. Variation of (**c**) hydrodynamic sizes and (**d**) zeta potentials of AuNPs with different concentrations of hydrogel.

Figure 3a,b shows the variation in sizes and zeta potentials of PEG-coated AuNPs with different concentrations of PEG. The suffix for each sample represents the concentration of PEG solution used for replacing the CTAB on the AuNPs. After coating, centrifugation was done to remove the unbound PEG from the solutions, indicating that the measurement data only show changes in size and zeta potential of the PEG-coated AuNPs. The DLS sizes for all the AuNPs except AuHex show an increasing trend in hydrodynamic sizes with an increase in initial concentration of PEG. This is further accompanied by reduction in zeta potential, representative of cationic CTAB being replaced by anionic thiolated PEG, owing to higher affinity of SH to Au [51]. It should however be noted that the AuNS are inherently negatively charged owing to citrate capping, indicated by negative zeta potential for AuNS without PEG coating. On the other hand, AuHex did not show appreciable changes in terms of hydrodynamic size as well as zeta potential upon coating with PEG. This can be attributed to the fact that the DLS measurements show the largest sizes for CTAB-coated AuHex among all the AuNPs studied. Since the sizes reported are intensity-weighted, larger particles show stronger scattering cross sections during DLS measurements, leading to an inability to detect minute changes in the surface layer owing to substitution of CTAB, a long 16 C chain surfactant, with PEG-SH, a macromolecule having a molecular weight of 5000 Da. Further uncertainty in the size measurements of AuHex results from the multiple facets observable in S(T)EM images (see Figure 1c), leading to multiple axes of rotation, which further introduces variations in the calculated diffusion coefficients based on spherical NPs. Figure 3c,d show the variation in sizes and zeta potentials of PEG-hydrogel coated AuNPs with different concentrations of hydrogel at a fixed PEG concentration of 0.5 mg/mL. PEG has been used in the study to replace the bound CTAB (cationic) atop the AuNPs. Although during screening experiments different concentrations of PEG were tried, here the concentration was fixed at 0.5 mg/mL. This was done as a significant reduction in the zeta potential for the coated NPs was achieved at this concentration, reflecting a successful replacement of CTAB at this concentration for all the AuNPs. Similar to the centrifugation step followed after PEG coating, the PEG-hydrogel-coated NPs were centrifuged in order to remove unbound hydrogel before performing the size and zeta potential measurements. Size measurements show an increase after hydrogel coating and with increase in hydrogel concentration. The hydrogel coating on the NPs is further supported by a decrease in the zeta potential values for all the shapes. Although experiments performed at different hydrogel concentrations show effective coating, concentrations of 1.67 mg/mL and 3.3 mg/mL were selected for performing Cyt *C* loading and release studies, in order to investigate the effect of hydrogel content in such NP systems. Higher concentrations were not used in the study as some of the shapes, namely AuHex and AuNS samples, had large hydrodynamic sizes while being coated with high concentrations of hydrogel (Figure 3c) and hence were not used for further loading and release experiments. 

**Figure 4:**
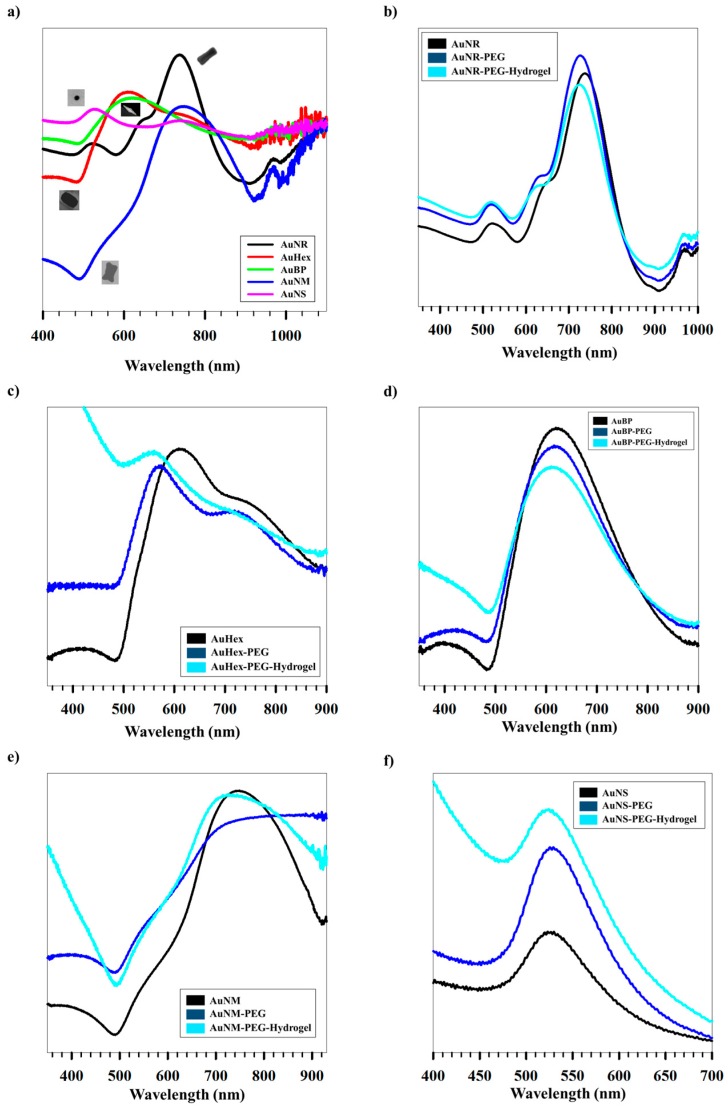

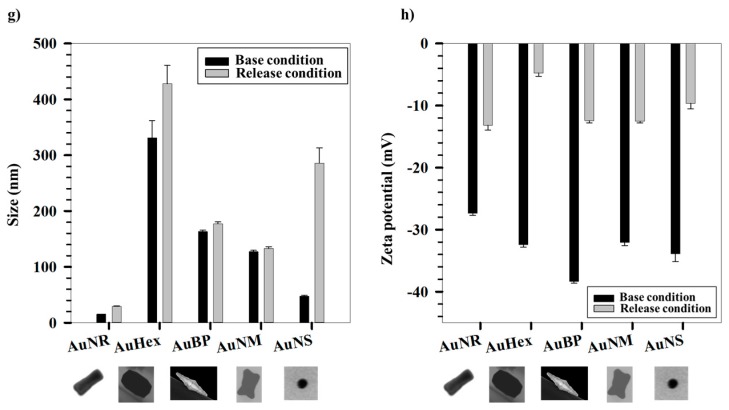
(**a**) UV-Vis spectra for differently shaped AuNPs. Variation of UV-Vis spectra after coating with PEG followed by hydrogel coating for (**b**) AuNR; (**c**) AuHex; (**d**) AuBP; (**e**) AuNM; and (**f**) AuNS; Variation of (**g**) hydrodynamic sizes and (**h**) zeta potentials of differently shaped AuNPs measured at base and release conditions, respectively.

Figure 4a shows the LSPR characteristics of the different AuNPs measured using UV-Vis spectrometry. AuNS show an LSPR peak at 523 nm, characteristic of spherical particles [43], whereas the anisotropic NPs AuHex, AuBP, and AuNM had their peaks at 609, 619 and 749 nm, respectively, as previously reported by our group [42]. On the other hand, AuNR showed two distinct peaks, the first corresponding to the transverse axis (522 nm) and the second corresponding to the longitudinal axis (737 nm). Figure 4b–e shows the changes in Uv-Vis spectra for AuNR, AuHex, AuBP, AuNM, and AuNS, respectively, upon coating with PEG (0.5 mg/mL), followed by hydrogel (3.3 mg/mL). AuNS shows a bathochromic shift from 523 to 529 nm upon coating with PEG, indicating a hydrophilic environment around the NPs, as has been reported earlier [43]. However, upon coating with hydrogel, the LSPR peak blue shifts (524 nm), likely due to absorption of water away from the NP surrounding by the hydrogel, result in a relatively water-deficient surrounding in the immediate vicinity of the NPs. In the case of the anisotropic NPs, a blue shift in the LSPR maxima is observed after PEG as well as hydrogel coating. A hypsochromic shift in the LSPR maximum upon PEG coating may be attributed to a gradual replacement of the CTAB bilayer with PEG, leading to uneven coating of PEG as a result of shape anisotropy. Furthermore, different coating densities on different facets of the anisotropic NPs, different conformations of the hydrogel guided by different shapes of the AuNPs or a combination also explain the shift. On subsequent coating with hydrogel, the observed blue shift is most likely due to the water absorption properties of the hydrogel leading to decreased water content adjacent to the AuNPs, as observed for spherical NPs as well. 

DLS and zeta potential measurements performed for all the samples at both base conditions (room temperature and pH at synthesis conditions) as well as release conditions (40 °C, 3 pH), as shown in Figure 4g,h, respectively, exhibit a swelling collapse behavior of the PEG-hydrogel coated AuNPs. AuNS show a maximum swelling efficiency of ~99% that is defined as the ratio of the volume change of the NPs between the base and release conditions to the volume at release condition. In contrast, the swelling efficiency for the anisotropic AuNPs decreases in the order AuNR > AuHex > AuBP > AuNM (Supporting Information). Our previous studies have shown that NPs reside on the periphery of the hydrogel units and act as cross-linkers, pulling the hydrogel matrix closer. The cross-linking density accounted for in such systems depends on the size, shape, particle number, and surface functionalization, among other factors. Herein, we observe that all the differently shaped AuNPs are located peripherally with respect to the hydrogel units for all the systems (see Figure 1). The particle concentrations used for the coating studies vary in the following order AuNM > AuNS > AuNR ≈ AuHex > AuBP (Table S1, Supporting Information), whereas the S(T)EM sizes vary as AuBP > AuHex > AuNM > AuNR > AuNS. Combining the particle concentrations with their sizes and inferring from the S(T)EM data of the NPs both before and after coating (see Figure 1), AuBP (large particles) have the lowest number of particles per unit volume in comparison to AuNS, AuNR, and AuNM, while AuHex has intermediate particle numbers. This results in higher number of particles for AuNS, AuNR, and AuNM cross-linking the gelling units, while fewer and bigger AuBP and AuHex act as bridge molecules connecting the gelling units. The VPTT of the pNIPAm-AAc hydrogels used in this study has been previously reported as 39 °C [52]. As a combined effect of temperature and pH, the individual hydrogel units collapse under release conditions. However, these collapsed hydrogel blocks are pulled together by bridging AuNPs of different shapes. As is evident from the S(T)EM images, several hydrogel units are bridged by a few large particles (AuBP and AuHex), whereas these units are cross-linked by a larger number of smaller NPs (AuNS, AuNR, and AuNM), resulting in higher flexibility of the collapsing network. This is exhibited in the high swelling efficiencies of the smaller NPs. (Figure S1, Supporting Information)

**Figure 5:**
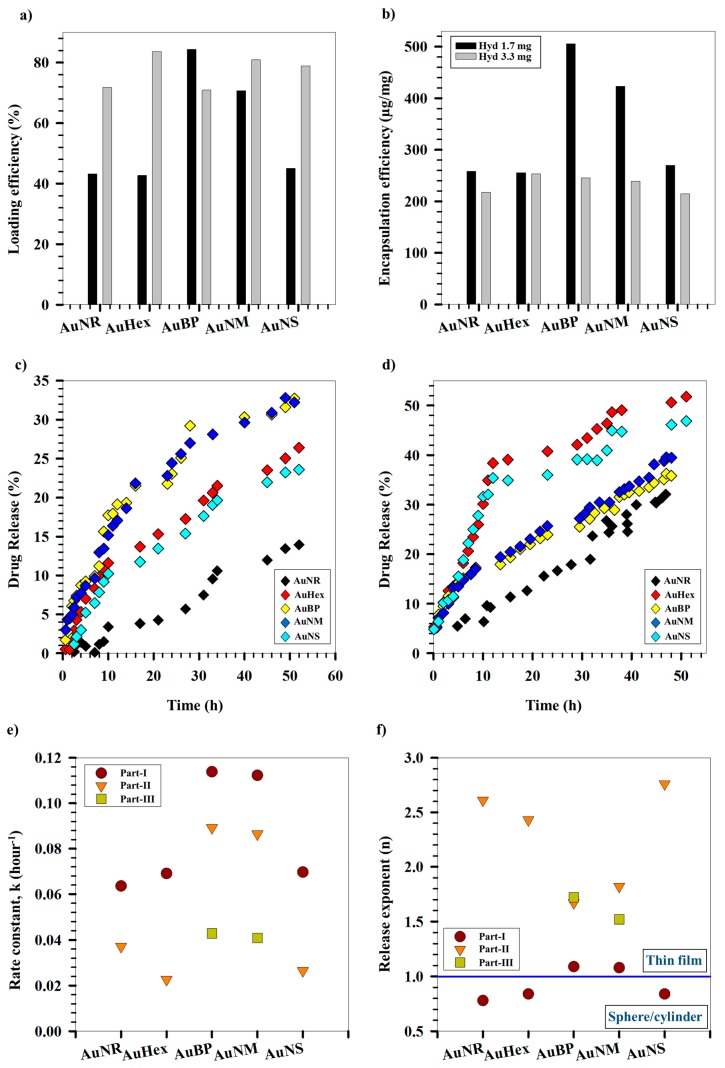
(**a**) Loading and (**b**) encapsulation efficiencies for differently shaped AuNPs for two different hydrogel concentrations. Release kinetics of Cyt *C* from differently shaped AuNPs for (**c**) 1.7 mg/mL and (**d**) 3.3 mg/mL hydrogel concentrations respectively. (**e**) Rate constants and (**f**) release exponents for Cyt *C* release from differently shaped AuNPs.

In order to understand the effect of the relative arrangement of the differently shaped AuNPs and hydrogel units and the relative amount of hydrogel on the loading and release of cargo molecule, the hydrogel-PEG-coated AuNPs were loaded with model protein drug Cyt *C*, using a modified breathing-in method. Figure 5a,b shows the loading and encapsulation efficiencies (LE and EE respectively) of the hydrogel-PEG-coated AuNPs (two different hydrogel concentrations, viz., 1.67 mg/mL and 3.3 mg/mL), respectively. All the systems show high loading efficiencies (LE, ~80%) for higher hydrogel concentrations. An immediate effect on the LE is observed upon decreasing the hydrogel concentration to 1.7 mg/mL. AuNR, AuHex and AuNS show LE ~40%, whereas LEs of AuBP and AuNM remain unchanged. LE gives an indication of the fraction of initial Cyt *C* loaded into the hydrogel-PEG-coated AuNPs. We observe a decrease in the LE of AuNR, AuHex, and AuNS with the decrease in the hydrogel concentration. This can be attributed to either an apparent increase in the initial concentration of the drug owing to the decrease in the hydrogel concentration or a lower binding of the drug to fewer hydrogel units. This further shows that the mode of loading of Cyt *C* into hydrogel-PEG-coated AuNPs is via interaction between Cyt *C* and the hydrogel units. Previous research has shown that the heme ligand, located in the lysine-rich region of Cyt *C*, interacts with the negatively charged hydrogel units via coulombic forces, resulting in polymer–protein complex formation [53,54]. On the other hand, there is not an appreciable change in the LEs for AuBP and AuNM with decrease in hydrogel concentration. As the AuBP has large sizes, it blocks the effective interaction sites of the hydrogel units with the protein, whereby the hydrogel concentration does not affect the LEs. However, for AuNM, a higher particle concentration as well as its intermediate size range (~hydrogel size) effectively hinders the absorption of Cyt *C*. However, the EEs of these systems decrease with increasing hydrogel concentration, indicating more available surface sites for efficient drug absorption. This is further supported by the fact that there is no observable change in EEs for AuNR, AuHex, and AuNS upon an increase in hydrogel concentration, explained by the increased amount of loaded drug in an apparently increased number of hydrogel units. 

Figure 5c,d shows the Cyt *C* release profiles from different AuNP systems with 1.67 and 3.3 mg/mL hydrogel coating, respectively. The release was measured via a time-dependent, dialysis-based approach over a period of 50 h using UV-Vis spectroscopy. The release studies have been conducted at 40 °C and pH 3, since at these conditions the hydrogels were observed to undergo maximal collapse as an effect of combined response to temperature and pH. The release data therefore indicate the most effective squeezing out of the drug under these conditions in response to external stimuli. An enhanced release of Cyt *C* is observed for AuNR, AuHex, and AuNS on increasing the hydrogel concentration used for coating the AuNPs. In contrast, no substantial change in the release percentage of Cyt *C* is evidenced for AuBP and AuNM. The release percentages follow the same order as LEs, indicating diffusion-based release governed by a Cyt *C* concentration gradient. However, a formidable increase in release percentage is seen for AuBP when comparing their release profiles with those of AuNR at higher hydrogel concentration (both having the same LEs). This may be attributed to the larger sizes of AuBP in addition to lower particle numbers compared to AuNR, leading to a greater openness of the hydrogel matrix for drug diffusion out of the matrix. A similar trend is noted for AuNS and AuHex, with the latter (having the highest LE) showing the maximum release percentage among all the systems studied. Although the LE of AuNM is comparable to that of AuNS (particle numbers higher than for AuNM), the AuNS act as efficient cross-linkers owing to their smaller sizes, pulling closer the collapsing hydrogel units. This creates more openness in the matrix, enabling more of the drug to escape into the release medium. Thus, the release of Cyt *C* from the AuNPs is guided by their sizes, shapes, particle concentrations, and efficiencies in acting as cross linkers among the hydrogel units under various release conditions, among other parameters.

Figure 5e,f shows the variation of rate constant and release exponent, respectively, for all the AuNP systems, measured at different time intervals throughout the release of Cyt *C*. The release domain for AuNR, AuHex, and AuNS was divided into two time regimes (Part I and Part II), whereas for AuBP and AuNM it was divided into three time regimes (Part I, Part II, and Part III) based on distinguishably different release zones over these time regimes. On fitting the data to different rate models, the systems showed a best fit with zero-order release kinetics. A decrease in the rate constant can be observed in all five AuNP systems with respect to increasing time. This is interpreted in terms of a temporal decrease in the concentration gradient of the drug inside the hydrogel-PEG-coated AuNPs and the bulk of the release medium. This determines the driving force for the diffusion of Cyt *C* out of the hydrogel matrix. 

Peppas’ equation, which relates the log of cumulative fraction of drug released (F) over time to the log of time [55], was used to determine the release geometry followed by different AuNP systems. It can be observed that all the AuNPs follow a spherical/cylindrical geometry in the first phase of the release, which transforms into a thin film as time progresses. This is indicated by a temporal transition of the release exponent (*n*), obtained from regression analysis of ln *F* versus ln *t* plots (see Figure S2, Supporting Information), from values close to 1 to values higher than 1. At the onset of release, the hydrogel-PEG-coated AuNPs behave mostly as spherical units squeezing out the drug, owing to the collapse of the individual hydrogel units. Owing to a cross-linking effect provided by the AuNPs, the rate of collapse of the hydrogel units is retarded by the presence of inorganic NPs in the matrix. This effect is dependent on the shape, size, and particle numbers and determines how effectively the spherical/cylindrical to thin film transition will occur. Higher values of release exponent (>1) indicate Super Case-II transport of Cyt *C*, a mode of drug release that combines diffusion-controlled and visco-elastic relaxation-controlled drug release [56,57].

## 3. Conclusions

We report the functionalization of five differently shaped AuNPs, nanorods (AuNR), tetrahexahedral (AuHex), bipyramids (AuBP), nanomakura (AuNM), and nanospheres (AuNS), with PEG followed by temperature- and pH-sensitive pNIPAm-AAc hydrogel. S(T)EM images revealed AuBP as the largest NPs, having an A.R. of 3.8 ± 0.5, while AuNS had the smallest size of 17.2 ± 2.2 nm. The successful coatings on the AuNPs were confirmed through the decrease in surface charge, coupled with an increase in hydrodynamic size and shifts in LSPR peaks observed through UV-Vis spectrometric measurements. The AuNPs were observed to act as cross-linkers between the hydrogel units, pulling together the collapsed units when subjected to high temperature (above VPTT) and acidic pH.

In order to investigate the effect of hydrogel content and particle shape and size on the loading and release of protein drugs, Cyt *C* was loaded into the systems using a modified breathing-in method. Higher loading efficiency was observed for all the samples with increased hydrogel content. However, the particle number, shape, size, and cross-linking effect of AuNPs were found to influence the loading efficiency as well as the release kinetics of Cyt *C*. Under the studied release conditions of high temperature (40 °C) and acidic pH (pH = 3), a higher release was seen with higher hydrogel content and larger AuNPs (AuHex). The NPs show a temporal transition from spherical/cylindrical geometry to thin films, indicating super case-II transport, which takes into account both diffusion- and visco-elastic relaxation-based release profiles. A combination of AuNPs with hydrogels exhibits optical and swelling collapse properties that enable multifunctionality in terms of imaging or tracking and drug delivery applications.

## 4. Materials and Methods

*O*-[2-(3-Mercaptopropionylamino)ethyl]-*O*′-methylpolyethylene glycol (PEG-SH) of molecular weight 5000 Da, *N*-isopropylacrylamide (NIPAm), *N*-*N*′-Methylene-bisacrylamide (BIS), Acrylic acid (AAc), potassium persulfate (KPS), Didecyldimethylammonium bromide (DDAB, 98%), Chloroauric acid (HAuCl_4_·3H_2_O, 99.999%), d-(−)-Isoascorbic acid (AsA, 98%), Sodium borohydride (NaBH_4_, ≥96%), and Cytochrome *C* (Cyt *C*) samples were purchased from Sigma-Aldrich^®^ (Schnelldorf, Germany). Cetyltrimethylammonium bromide (CTAB, 99%+) and Sodium citrate dihydrate (Na-citrate, ACS grade) were obtained from Acros Organics^®^ (Geel, Belgium) and Merck^®^ (Darmstadt, Germany), respectively. Oleic acid (OA, 90%) was purchased from Alfa Aesar (Karlsruhe, Germany). All the chemicals were used as received without any further purification or modification. All solutions were prepared using distilled de-ionized water (MQ-water), having a resistivity ~18.2 MΩ/cm at 25 OC, taken from Simplicity^®^ Millipore (Darmstadt, Germany ) water purification system.

### 4.1. Synthesis of Hydrogel

pNIPAm-AAc hydrogel was synthesized using our previously reported method that employs precipitation polymerization [53]. In a typical synthesis, 181 mg (1.6 mmol) of NIPAm was added along with 14.8 mg (0.07 mmol) of cross-linker, BIS, in a round-bottomed flask, maintained at 70 °C. Thereafter, 10 mL of 4.2 mM of SDS was added to the mixture. The solution was degassed under a nitrogen atmosphere for one hour, after which 126 µL of 1.46 M AAc was added. The monomer, co-monomer, and cross-linker molar ratio were maintained at 1.6:0.1:0.2. Four hundred microliters of 103.6 mM KPS were added to initiate the precipitation polymerization reaction. The reaction was allowed to continue at 70 °C under a nitrogen atmosphere for a further 2 h. 

### 4.2. Synthesis of Spherical AuNPs

A modified Turkevich approach was used for the synthesis of spherical AuNPs (AuNS) [43]. In a typical synthesis, 10 mL of 1.74 mM HAuCl_4_ were added dropwise to 10 mL of 10 mM sodium citrate in a 25-mL round-bottomed flask maintained at 70 °C. The reaction was maintained at a temperature of 70 °C and allowed to continue for 20 min. Thereafter, the solution was cooled to room temperature and AuNS were separated from the reaction solution using centrifugation at 14,500 rpm for 20 min. The supernatant was removed and the particles were re-dispersed in 5 mL MQ-water.

### 4.3. Synthesis of Au Seed

Five milliliters of 0.5 mM HAuCl_4_ were added to 5 mL of 0.2 M CTAB in a 25-mL round-bottomed flask under stirring for 7 min. Then 1.6 mL of freshly prepared 3.75 mM NaBH_4_ solution were added to the reacting mixture with further stirring for 2 min. Thereafter, stirring was stopped and the reaction was allowed to continue for 30 min.

### 4.4. Synthesis of Anisotropic AuNPs

The Ag-assisted seeded growth method was utilized for the synthesis of anisotropic AuNPs, similar to a strategy reported previously by our group that involves CTAB-coated Au seeds, followed by a one-step seeded growth in the presence of Ag [58]. For the synthesis of Au nanomakura (AuNM), an intermediate growth solution is used instead of the CTAB capped seed solution. 

In a typical synthesis, 15 mL of an aqueous solution of CTAB and co-surfactant, in various ratios, were made at 110 °C, as reported in Table 2, with constant stirring at 500 rpm. The solution was allowed to cool down to 35 °C and 750 µL of 4 mM AgNO_3_ were added to the solution. The solution was allowed to stand under stirring for 15 min, following which 15 mL of 1 mM HAuCl_4_·3H_2_O were added. After 15 min of adding HAuCl_4_·3H_2_O, the stirring speed was increased to 1000 rpm and 135 µL of 128 mM AsA were added, which rendered the yellow solutions colorless. Thereafter, 96 µL of seed solution were added to AuNR, AuHex, AuBP, and an intermediate growth solution (A*). Then 300 µL of A* was added to AuNM. The stirring was stopped and the reaction was allowed to continue for 24 h at 35 °C. The particles were separated by centrifugation and re-dispersed in 5 mL MQ-water.

### 4.5. Coating of AuNPs

In order to replace the cationic CTAB coating, the NPs were subsequently coated with PEG. Furthermore, to determine the temperature and pH response to the AuNP systems, hydrogel was coated onto the already PEG-functionalized AuNPs. 

To investigate the effectiveness of CTAB replacement by PEG, three different PEG concentrations were selected (0.5, 1 and 2 mg/mL). In a typical coating experiment, 400 µL of AuNP solution were mixed with 1 mL of PEG solution and stirred at 500 rpm for 2 h in a final volume of 2 mL. Thereafter, in order to remove unbound PEG, the solutions were centrifuged and the NPs were re-dispersed in 3 mL MQ-water. To coat the NP systems with hydrogel, different weights of hydrogel (1.67, 3.3 and 6.6 mg) were added to the above solution and left to stir at 500 rpm for 2 h. The solutions were centrifuged to remove the unbound hydrogel and the NPs were re-dispersed in 3 mL MQ-water. 

### 4.6. Loading and Release with Cyt C

Cyt *C* was loaded using a modified breathing-in mechanism, as reported previously [53]. For this study, two samples each of hydrogel-PEG-coated NPs were taken in separate 1.5-mL Eppendorf tubes for centrifugation at 14,000 rpm for 20 min. The supernatant was removed and 1 mL of 0.5 mg/mL Cyt *C* was added to each sample. The samples were then put on shaking for 2 h. The reaction was stopped and the two samples of each NPs were mixed together to form a 2-mL loaded solution. The loaded solutions from five AuNP systems were put on dialysis overnight in order to remove any free Cyt *C* from the system. After dialysis, the samples were removed and taken further for release studies. Release of Cyt *C* was studied using dialysis setup and monitored using UV-Vis spectroscopy. All the release studies were conducted under conditions of 40 °C and pH 3, with the pH of the release medium being maintained using 1 M HCl.

$$L.E.=\left(\frac{C_{\mathrm{cyt},0}-C_{\mathrm{cyt},t}}{C_{\mathrm{cyt},0}}\right)*100\%$$

$$E.E.=\left(\frac{C_{\mathrm{cyt},0}*L.E.}{100*C_{\mathrm{hyd}}}\right)$$

where *C*_cyt,0_ is the concentration (mg/mL) of Cyt *C* at the start of loading, *C*_cyt,t_ is the final concentration (mg/mL) of Cyt *C* after loading, and *C*_hyd_ is the concentration (mg/mL) of the hydrogel. The Cyt *C* concentrations were determined by the absorbance method.

### 4.7. Characterization Techniques

#### 4.7.1. Scanning (Transmission) Electron Microscopy (S(T)EM)

Bright field (BF) S(T)EM images were acquired using a Hitachi S-5500 electron microscope (Hitachi High-Technologies Europe GmbH, Mannheim, Germany) operating at 30 kV accelerating voltage. TEM images were obtained in BF mode. TEM grids were prepared by placing several drops of the solution on a Formvar carbon-coated copper grid (Electron Microscopy Sciences) and wiping immediately with Kimberly-Clark Kimwipes to prevent further aggregation owing to evaporation at room temperature.

#### 4.7.2. Dynamic Light Scattering (DLS)

The size distribution and zeta potential of the NPs were measured using a Malvern Zetasizer Nano-ZS instrument (Malvern Instruments Ltd., Worcestershire, UK) and the manufacturer’s own software. The solvent used for the AuNPs was MQ water.

#### 4.7.3. Ultraviolet-Visible Spectroscopy (UV-Vis)

UV-Vis spectra were acquired with a UV-2401PC (Shimadzu Corporation, Duisburg, Germany) spectrophotometer. The spectra were collected over the spectral range 200–1100 nm.

## Figures and Tables

**Table 1:** Differences in the sizes obtained from S(T)EM and DLS measurements.

AuNPs	Aspect Ratio (avg ± st. dev)	S(T)EM Size (nm) Length/Width	DLS Size (nm)
AuNR	3.4 ± 0.5	42 ± 4/13 ± 2	14.4 ± 0.7
AuHex	1.4 ± 0.2	233 ± 25/171 ± 20	164.8 ± 5.6
AuBP	3.8 ± 0.5	382 ± 107/107 ± 45	144.9 ± 1.1
AuNM	1.5 ± 0.4	118 ± 15/83 ± 14	97.4 ± 2.4
AuNS	–	17 ± 2	22.5 ± 0.4

**Table 2:** Moles of surfactant and co-surfactant used for synthesis of AuNPs.

Sample Name	Co-Surfactant	Moles of CTAB	Moles of Co-Surfactant
**AuNR**	Oleic acid	3.3 × 10^−3^	6.3 × 10^−5^
**AuHex**	Oleic acid	3.3 × 10^−3^	9.4 × 10^−4^
**AuBP**	DDAB	3.3 × 10^−3^	4.3 × 10^−4^
**AuNM**	Oleic acid	3.3 × 10^−3^	9.4 × 10^−5^
A*	Oleic acid	3.3 × 10^−3^	9.4 × 10^−5^

## Supplementary Materials

The following are available online at www.mdpi.com/2310-2861/3/4/42/s1, Figure S1: Swelling efficiencies of different shaped AuNPs, Figure S2: ln(*F*) vs. ln(*t*) plots for AuNR (a) Part-I; (b) Part-II; AuHex (c) Part-I; (d) Part-II; AuBP (e) Part-I; (f) Part-II; (g) Part-III; AuNM (h) Part-I; (i) Part-II; (j) Part-III; AuNS (k) Part-I; (l) Part-II. Table S1: Physico-chemical properties of different shaped AuNPs.
