# Supplementary material for: The Influence of Differently Shaped Gold Nanoparticles Functionalized with NIPAM-Based Hydrogels on the Release of Cytochrome C
Source: Gels. 2017 Nov 8;3(4):42. doi: 10.3390/gels3040042 (PMC6318608; doi:10.3390/gels3040042)
Supplement: Supplementary file 1 [file gels-03-00042-s001.pdf]

The Influence of Differently Shaped Gold Nanoparticles Functionalized with NIPAM-Based Hydrogels on the Release of Cytochrome *C*

Sulalit Bandyopadhyay, Anuvansh Sharma and Wilhelm Robert Glomm

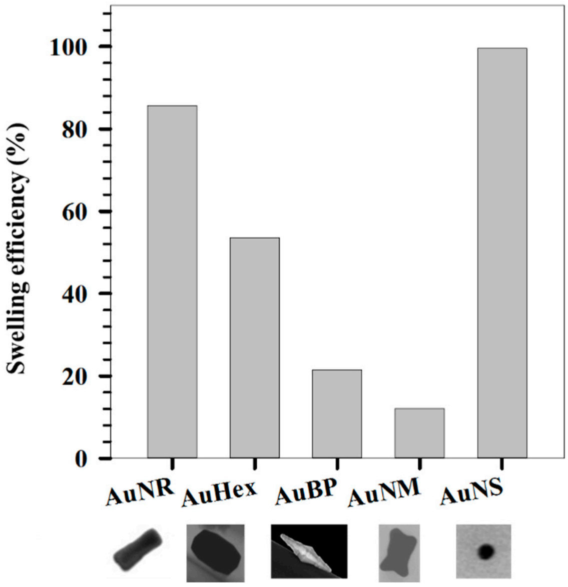

Figure S1. Swelling efficiencies of different shaped AuNPs.

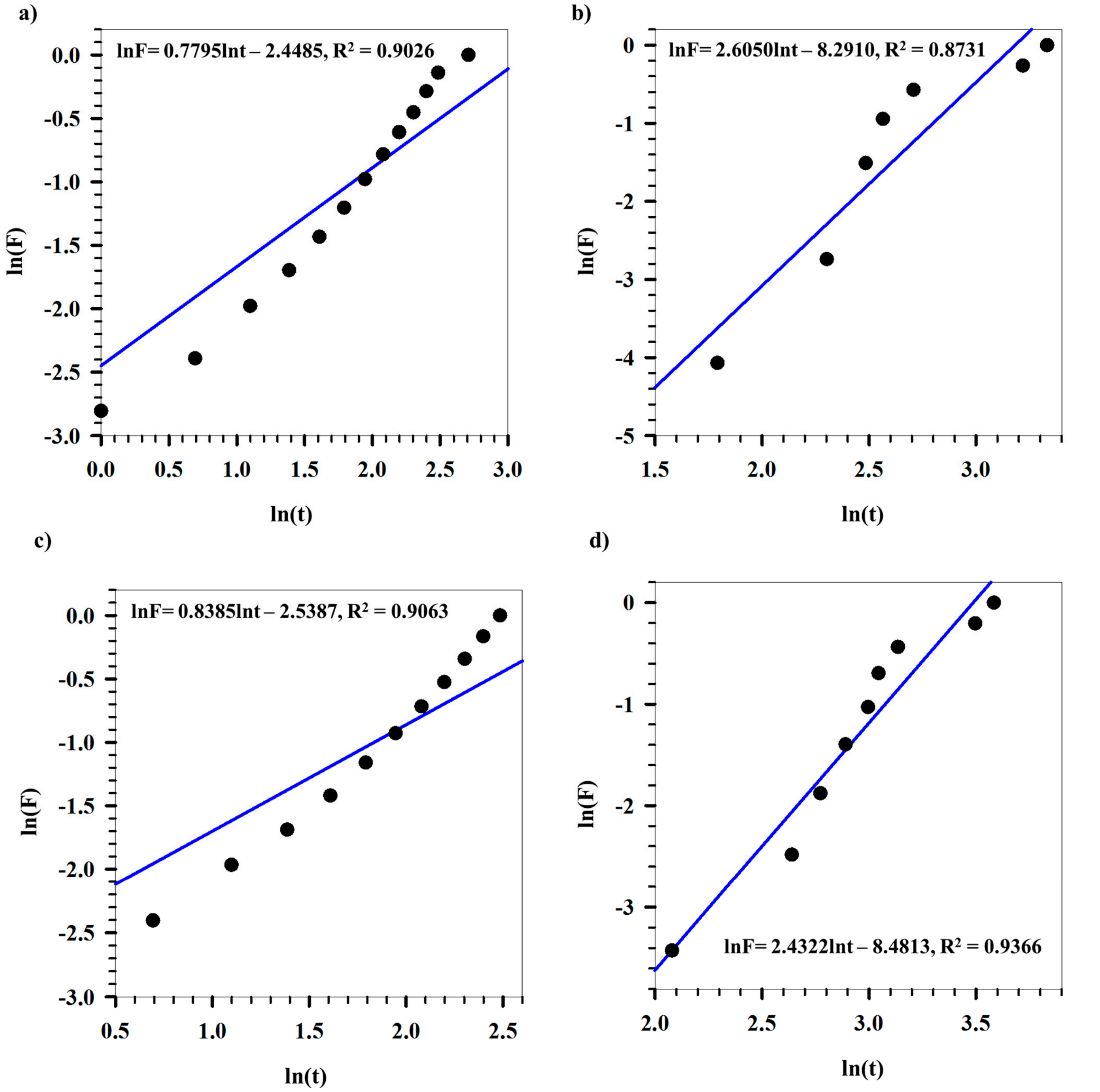
a)
lnF= 0.7795lnt – 2.4485, R² = 0.9026
ln(F)
ln(t)
b)
lnF= 2.6050lnt – 8.2910, R² = 0.8731
ln(F)
ln(t)
c)
lnF= 0.8385lnt – 2.5387, R² = 0.9063
ln(F)
ln(t)
d)
lnF= 2.4322lnt – 8.4813, R² = 0.9366
ln(F)
ln(t)

e)

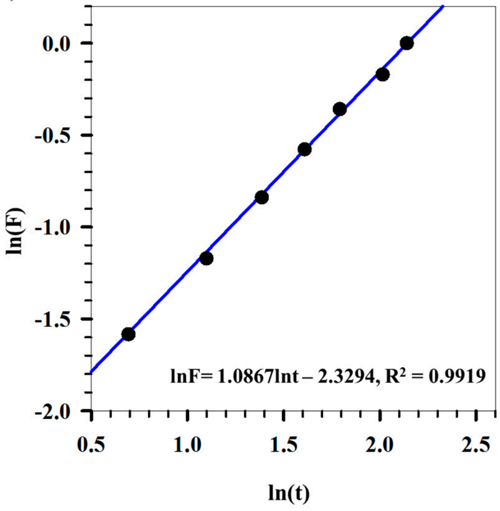
lnF= 1.0867lnt – 2.3294, R² = 0.9919
ln(F)
ln(t)

f)

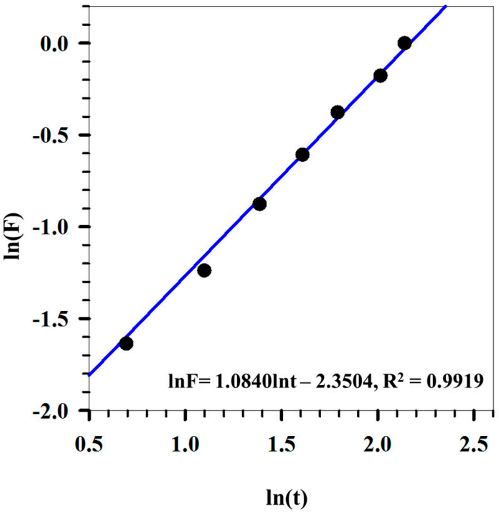
lnF= 1.0840lnt – 2.3504, R² = 0.9919
ln(F)
ln(t)

g)

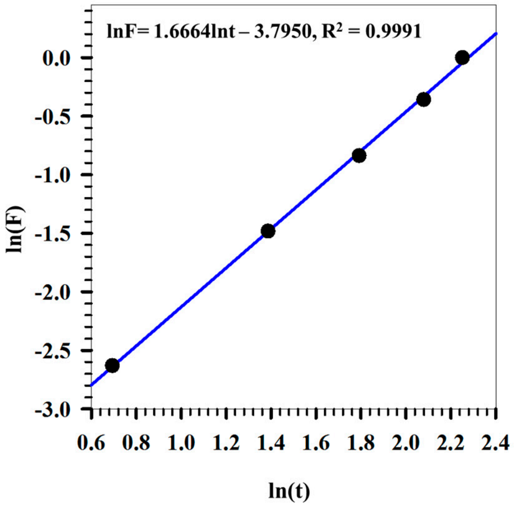
lnF= 1.6664lnt – 3.7950, R² = 0.9991
ln(F)
ln(t)

h)

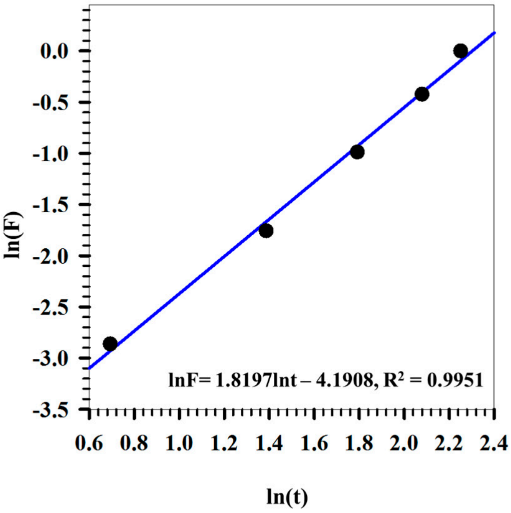
lnF= 1.8197lnt – 4.1908, R² = 0.9951
ln(F)
ln(t)

i)

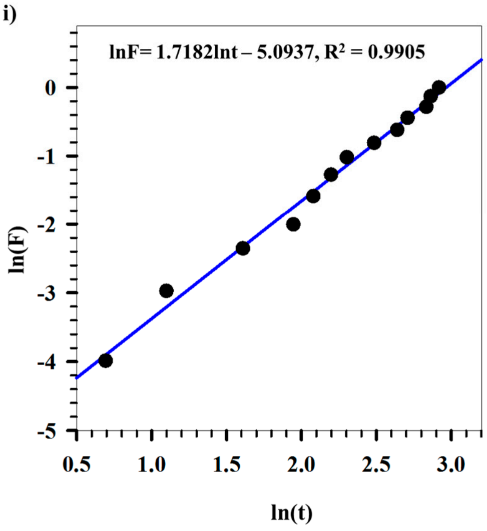
lnF= 1.7182lnt – 5.0937, R² = 0.9905
ln(F)
ln(t)

j)

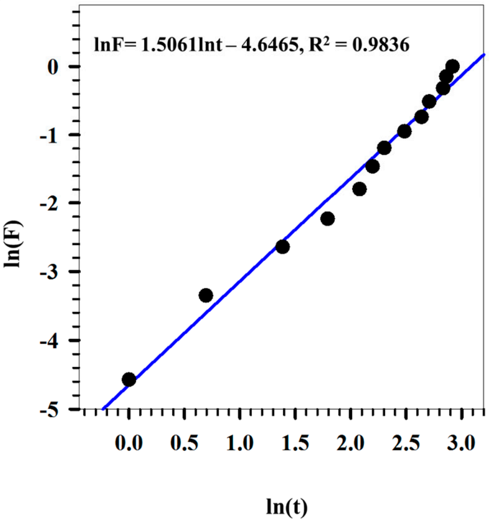
lnF= 1.5061lnt – 4.6465, R² = 0.9836
ln(F)
ln(t)

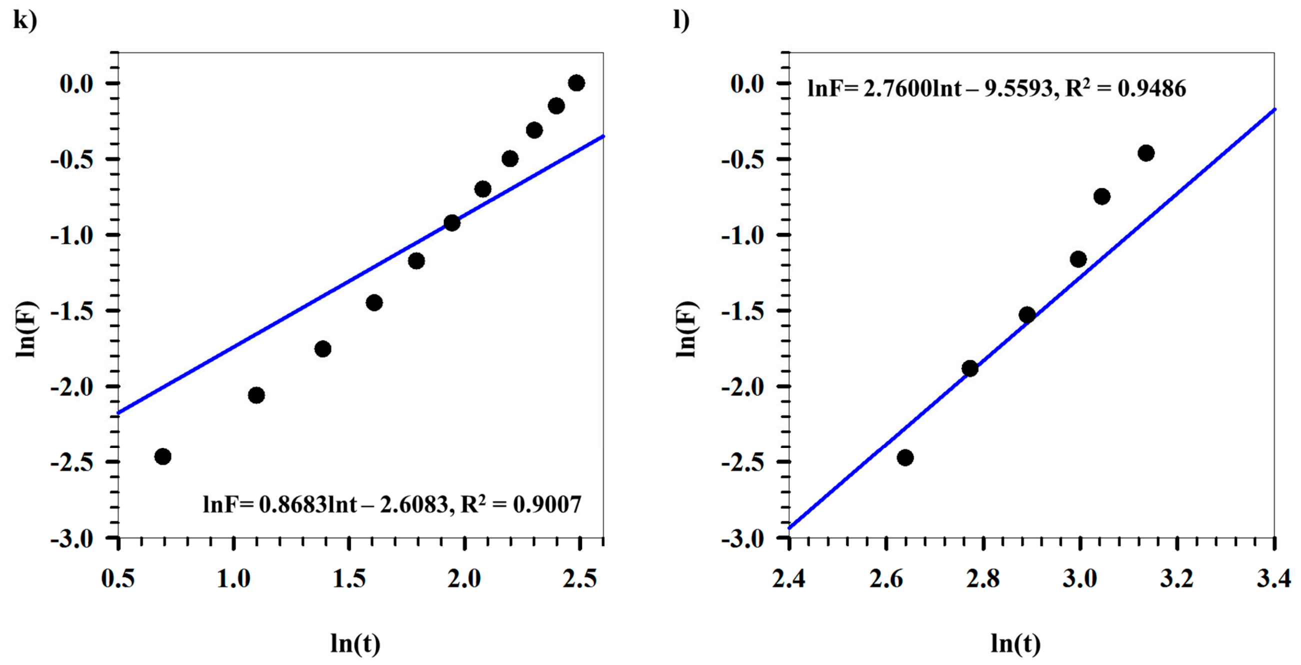

Figure S2. ln(F) vs ln(t) plots for AuNR **a**) Part-I, **b**) Part-II, AuHex **c**) Part-I, **d**) Part-II, AuBP **e**) Part-I, **f**) Part-II, **g**) Part-III, AuNM **h**) Part-I, **i**) Part-II, **j**) Part-III, AuNS **k**) Part-I, **l**) Part-II.

Table S1. Physico-chemical properties of different shaped AuNPs.

	**Particle Concentration (mg/mL)**	**Size (nm)**		**Particle Number (N)**	**Zeta Potential (mV)**	**Surface Charge Density (mV/m²)**
		Length	Width			
**AuNR**	23.3	41.8	12.6	3.95E+12	-27.4	-1.25E+15
**AuHex**	23.7	232.6	170.7	2.32E+10	-32.4	-4.77E+13
**AuBP**	19.1	381.7	106.5	4.25E+09	-33.9	-1.85E+13
**AuNM**	28.8	117.7	83.2	2.19E+11	-38.4	-2.2E+14
**AuNS**	25.0	17.2	17.2	6.07E+13	-32.1	-8.63E+15
